# The effect of Ramadan fasting during pregnancy on perinatal outcomes: a systematic review and meta-analysis

**DOI:** 10.1186/s12884-018-2048-y

**Published:** 2018-10-25

**Authors:** Jocelyn D. Glazier, Dexter J. L. Hayes, Sabiha Hussain, Stephen W. D’Souza, Joanne Whitcombe, Alexander E. P. Heazell, Nick Ashton

**Affiliations:** 1 Maternal and Fetal Health Research Centre, Division of Developmental Biology and Medicine, Faculty of Biology, Medicine and Health, University of Manchester, 5th Floor (Research), St Mary’s Hospital, Oxford Road, Manchester, M13 9WL UK; 2 Trust Library, Central Manchester University Hospitals NHS Foundation Trust, Education South, Oxford Road, Manchester, M13 9WL UK; 3 Division of Cardiovascular Sciences, Faculty of Biology, Medicine and Health, University of Manchester, 3rd Floor Core Technology Facility, 46 Grafton Street, Manchester, M13 9NT UK

**Keywords:** Birth weight, Fasting, Placenta, Pregnancy, Ramadan

## Abstract

**Background:**

Although exempt, many pregnant Muslim women partake in the daily fast during daylight hours during the month of Ramadan. In other contexts an impoverished diet during pregnancy impacts on birth weight. The aim of this systematic review was to determine whether Ramadan fasting by pregnant women affects perinatal outcomes. Primary outcomes investigated were perinatal mortality, preterm birth and small for gestational age (SGA) infants. Secondary outcomes investigated were stillbirth, neonatal death, maternal death, hypertensive disorders of pregnancy, gestational diabetes, congenital abnormalities, serious neonatal morbidity, birth weight, preterm birth and placental weight.

**Methods:**

Systematic review and meta-analysis of observational studies and randomised controlled trials was conducted in EMBASE, MEDLINE, CINAHL, Web of Science, Google Scholar, the Health Management Information Consortium and Applied Social Sciences Index and Abstracts. Studies from any year were eligible. Studies reporting predefined perinatal outcomes in pregnancies exposed to Ramadan fasting were included. Cohort studies with no comparator group or that considered fasting outside pregnancy were excluded, as were studies assuming fasting practice based solely upon family name. Quality of included studies was assessed using the ROBINS-I tool for assessing risk of bias in non-randomised studies. Analyses were performed in STATA.

**Results:**

From 375 records, 22 studies of 31,374 pregnancies were included, of which 18,920 pregnancies were exposed to Ramadan fasting. Birth weight was reported in 21 studies and was not affected by maternal fasting (standardised mean difference [SMD] 0.03, 95% CI 0.00 to 0.05). Placental weight was significantly lower in fasting mothers (SMD -0.94, 95% CI -0.97 to  -0.90), although this observation was dominated by a single large study. No data were presented for perinatal mortality. Ramadan fasting had no effect on preterm delivery (odds ratio 0.99, 95% CI 0.72 to 1.37) based on 5600 pregnancies (1193 exposed to Ramadan fasting).

**Conclusions:**

Ramadan fasting does not adversely affect birth weight although there is insufficient evidence regarding potential effects on other perinatal outcomes. Further studies are needed to accurately determine whether Ramadan fasting is associated with adverse maternal or neonatal outcome.

**Electronic supplementary material:**

The online version of this article (10.1186/s12884-018-2048-y) contains supplementary material, which is available to authorized users.

## Background

During the month of Ramadan, healthy adult Muslims abstain from eating and drinking from sunrise until sunset. This represents a form of intermittent fasting where both the quantity and quality of food eaten are altered [1]. Although pregnant Muslim women are exempt from fasting, evidence suggests that up to 90% partake in Ramadan fasting for at least part of the month [2, 3], being keen to share the cultural experience with their families. The estimate of 230 million Muslim women of childbearing age worldwide [4], with a fertility rate averaging 3.1 children per woman [4], leads to the potential for up to 535 million babies in each generation to be exposed *in utero* over Ramadan to a repeated cyclical pattern of maternal intermittent fasting.

Exposure to a restricted or sub-optimal diet during pregnancy affects fetal development and has life-long health impacts on the offspring [5]. Low birth weight and altered neonatal growth trajectories are associated with an increased risk of cardiovascular disease, diabetes [5], obesity [6] and impaired cognitive function [7]. Preterm delivery and reduced birth weight are more prevalent in women who eat less frequently while pregnant [8], suggesting that pregnant women who fast during Ramadan may be more likely to give birth to premature or underweight babies.

Although the impact of Ramadan fasting during pregnancy on the health of the child has been investigated [9–13], individual studies show conflicting results and sample sizes are often too small to allow evaluation of serious, but infrequent, outcomes. Furthermore, the timing of exposure to maternal fasting during Ramadan may affect the outcome [14], yet the trimester of fetal exposure to fasting is generally poorly defined in studies. Although fasting could arise at any pregnancy stage, occurrence early in the first trimester seems most likely as the mother may be unaware that she is already pregnant. Fasting during the first trimester has been reported to be associated with reduced birth weight [15], whereas placental weight, another predictor of health outcomes in offspring [16], is reportedly lower if the mother fasted during the second or third trimester [17].

Muslim women may seek advice from health practitioners regarding the safety of Ramadan fasting; however the current information available to pregnant women is contradictory [18] and clear guidance is lacking. Therefore, available evidence regarding associations between Ramadan fasting and pregnancy outcomes needs to be evaluated.

The aim of this systematic review and meta-analysis was to determine the effects of maternal intermittent fasting during Ramadan on a range of pregnancy outcomes.

## Methods

The systematic review and meta-analysis is reported in accordance with PRISMA guidelines [19]; the review protocol was registered with the International Prospective Register of Systematic Reviews (PROSPERO) on 8 July 2016 (CRD42016041949).

### Eligibility criteria, information sources, search strategy

Searches were carried out in EMBASE, MEDLINE, CINAHL, Web of Science, Google Scholar, the Health Management Information Consortium (HMIC) and Applied Social Sciences Index and Abstracts. In order to reduce publication bias, searches were also carried out in the Centre for Reviews and Dissemination databases, ProQuest and EThOS to uncover any relevant unpublished studies and grey literature. Reference lists of eligible studies were checked for other potentially eligible studies for inclusion. The search was not limited by dates but was limited to English-only publications. All searches were updated on 11 April 2018. See Additional file 1 for the EMBASE search strategy. Searches were performed by JW, SH and DH.

We included observational studies which reported either primary or secondary outcomes in pregnancies that were exposed to intermittent fasting during Ramadan compared to unexposed pregnancies. Randomised controlled trials or cluster randomised controlled trials were also eligible. Cohort studies with no comparator group (which only reported an outcome of interest in women who fasted during pregnancy) were excluded. If studies assumed fasting practice based solely upon ethnic group or family name then they were excluded as this was deemed to be unreliable. Studies were not excluded based on their geographical location or the timing of fasting with regard to trimester of pregnancy.

Studies were included if they reported a relevant pregnancy outcome in women who intermittently fasted during their pregnancy. The exposure of interest was intermittent fasting during the month of Ramadan during any stage of pregnancy. Studies looking at fasting during any other time period (prior to conception, postnatal period) were excluded.

Primary outcomes for this study were: perinatal mortality (the death of a baby before birth or during the first week of life), preterm birth (before 37 weeks of pregnancy) and small for gestational age (SGA) infants (as defined by each study or below the tenth centile for gestational age). Secondary outcomes were: stillbirth (the death of a baby before birth after 20 weeks’ gestation), neonatal death (the death of a baby during the first 28 days of life), maternal death (the death of the mother during pregnancy or the first 6 weeks postnatally), hypertensive disorders of pregnancy, gestational diabetes, congenital abnormalities (structural abnormalities of the fetus), serious neonatal morbidity, birth weight (continuous variable), low birth weight (< 2500 g), very low birth weight (< 1500 g), extremely preterm birth (< 28 weeks gestation) and placental weight (continuous variable).

### Data extraction

After removal of duplicates, all citations were screened for relevance using the full citation, abstract and indexing terms. Relevant studies were assessed for eligibility by two out of four reviewers (SH, DH, JG and SDS) according to the pre-specified inclusion and exclusion criteria, and where possible full manuscripts were obtained. Final decisions were made by two reviewers independently and a third (AH or NA) consulted to resolve any issues where necessary. Where data were missing or incomplete, attempts were made to contact the authors for clarification.

### Assessment of risk of bias

Included studies were assessed using the Risk Of Bias In Non-randomised Studies – of Interventions (ROBINS-I) tool [20], which categorises risk of bias as low, moderate, serious, critical and unclear, and the risk of bias category for each study was reported; if a study’s risk of bias was categorised as serious, critical or unclear, the effect of removing this study was tested and the relevant outcome(s) reported.

### Data synthesis

Meta-analysis was performed in STATA (Version 14) [21] using the *metan* [22] and *metabias* [23] commands. Random effects meta-analysis was used in anticipation of heterogeneity due to differences in study design.

For continuous variables (birth weight and placental weight), standardised mean differences (SMD) (Hedges’ g) with 95% confidence intervals were calculated. For binary variables (low birth weight and preterm delivery), odds ratios and 95% confidence intervals were calculated. The *I*^*2*^ statistic was calculated; this is derived from Cochran’s chi-squared statistic *Q* and is used to describe the percentage of between-study variation that is attributable to variability in the true exposure effect [24]. An *I*^*2*^ value of 0–30% was classified as low, 31–60% as moderate, 61–90% substantial and 91–100% considerable [25]. Funnel plots were created to test for small-study effects.

Where studies presented continuous data grouped by trimester in which fasting took place, length of fasting or stratified by other measures (e.g. fetal sex), then averages were taken to obtain overall means and standard deviations. Where outcome data were available by fasting trimester then data were stratified by trimester and the effect of this was investigated.

## Results

### Study selection

**Fig. 1:**
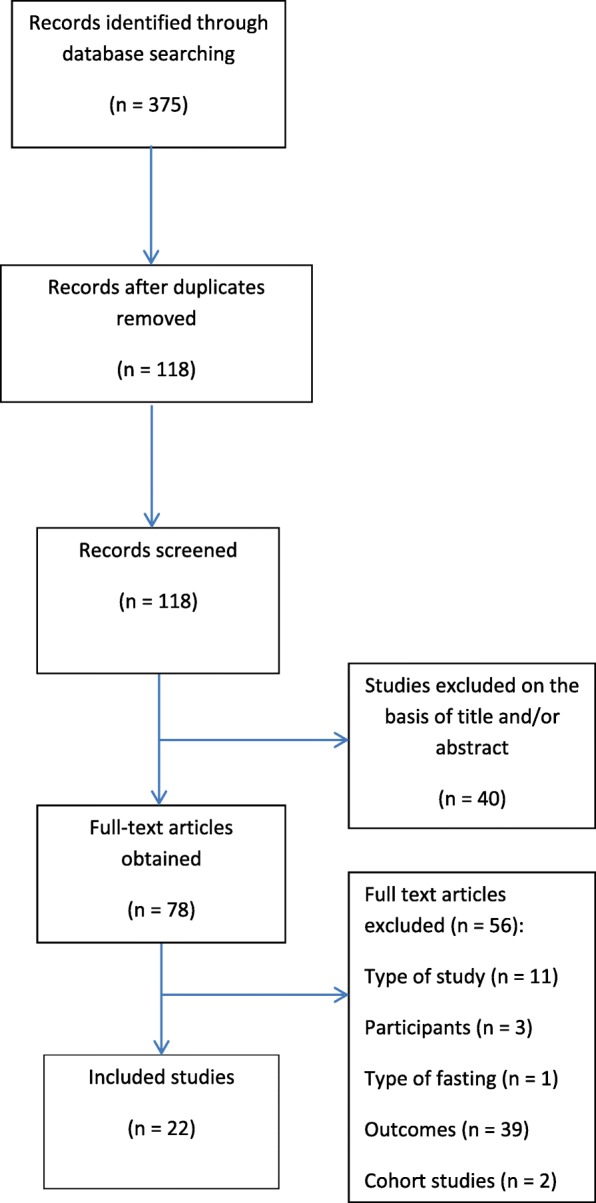
PRISMA diagram of included studies. Flow chart showing study selection

The search strategy identified 375 records (Fig. 1). After duplicates were removed 118 papers were screened on the basis of their titles and abstracts. Forty papers were excluded on this basis, resulting in 78 papers to be evaluated using their full text. After exclusions, 22 studies of 31,374 pregnancies were included in the final analysis.

### Study characteristics

Seven studies reported data for at least one of the co-primary outcomes (perinatal mortality, SGA infants and preterm birth) and all but one study [9] reported data on at least one secondary outcome (Table 1). Six studies were judged to be at moderate risk of bias; the other 16 were determined to be at low risk (Table 2). Heterogeneity for outcomes ranged from 0 to 98.5%.

**Table 1:** Characteristics of included studies

Authors	Year	Location and study period	Total pregnancies	Cases	Controls	Primary outcomes	Secondary outcomes	Trimester of fasting	Length and duration of fasting (days and average hours/day)	Risk of bias (ROBINS-I)
Alwasel	2011	Saudi Arabia, August 2000–April 2009	17,626	13,220	4406	–	BW, PW	1st, 2nd, 3rd	n/a	Moderate
Arab	2001	Iran, 1999	4343	3086	1257	SGA	BW, LBW	n/a	1 to 20+ days n/a	Moderate
Awwad	2012	Lebanon, September 2008	402	201	201	PTB	BW, LBW	1st, 2nd, 3rd	22 +/− 9 days n/a	Low
Azizi	2004	Iran, April–September 2001	191	98	93	–	BW	3rd	28 +/− 2 days 13 h20 min	Low
Bayoglu Tekin	2016	Turkey, June–July 2014	48	23	25	–	BW	3rd	18.2 +/− 2 days 17.24 h	Low
Daley	2017	United Kingdom, March 2007 – December 2010	5156	479	4677	PTB	BW, LBW	1st	n/a	Moderate
Hefni	1993	Egypt, 1991	322	167	155	–	BW, SB	3rd	n/a > 10 h	Low
Hızlı	2012	Turkey, August–September 2010	110	56	54	–	BW	3rd	12.9 +/− 2.5 days 15.3 h (12–19)	Low
Karateke	2016	Turkey, June–July 2014	240	120	120	–	BW, LBW	1st, 2nd, 3rd	n/a n/a	Low
Kavehmanesh	2004	Iran, January–September 2000	539	284	255	PTB	BW	n/a	24 +/− 9 days 13 h	Low
Makvandi	2013	Iran, 2013	300	150	150	–	BW, LBW	3rd	16.6+/−13.2 days n/a	Low
Malhotra	1989	United Kingdom, April–May 1987	22	11	11	SGA	BW, CA, NND, PW	3rd	n/a 7 h	Moderate
Naderi	2004	Iran, November 2001	101	51	50	–	BW	n/a	20+ days n/a	Low
Ozturk	2011	Turkey, September 2008	72	42	30	PTB	–	2nd	10+ days Approximately 12 h	Low
Petherick	2014	United Kingdom, October–December 2010	300	128	172	PTB	BW, GD, Hyp, LBW	1st, 2nd, 3rd	1–29 days Up to 18 h	Moderate
Rezk	2016	Egypt, June–July 2015	450	210	240	–	BW	3rd	30 days 12-16 h	Low
Sakar	2016	Turkey, August–October 2013	338	168	170	–	BW, PW	3rd	25.17 +/− 5.44 days n/a	Low
Sarafraz	2014	Iran, 2008	293	200	93	–	BW	1st, 2nd, 3rd	1 to 20+ days n/a	Low
Savitri	2018	Indonesia, July 2012 – July 2014	139	110	29	–	BW	1st, 2nd, 3rd	1–30 days n/a	Moderate
Seckin	2014	Turkey, July–August 2013	169	82	87	–	BW	3rd	23 +/− 3.6 days 18.7 h (17–20)	Low
Shahgheibi	2005	Iran, (dates unknown)	163	63	100	–	BW, LBW	3rd	10 to 20+ days n/a	Low
Ziaee	2010	Iran, October 2004	189	123	66	–	BW, LBW	1st, 2nd, 3rd	1 to 20+ days 13 h	Low

Primary outcomes: *SGA* small for gestational age, *PTB* preterm birth. Secondary outcomes, *BW* birth weight, *CA* congenital abnormalities, *GD* gestational diabetes, *Hyp* hypertension, *LBW* low birth weight, *NND* neonatal death, *PW* placental weight, *SB* stillbirth

**Table 2:** Risk of bias of included studies

Author	Year	Bias domain						
		Bias due to confounding	Bias in selection of participants into the study	Bias in classification of exposure	Bias due to missing data	Bias in measurement of outcomes	Bias in selection of the reported result	Overall
Alwasel	2011	**Low/Moderate**	Low	**Serious**	Low	Low	Low	**Moderate**
Arab	2001	Low	Low	Low	**Serious**	Low	Low	**Moderate**
Awwad	2012	**Moderate**	Low	Low	Low	Low	Low	**Low**
Azizi	2014	Low	Low	Low	**Moderate**	Low	Low	**Low**
Bayoglu Tekin	2016	Low	Low	Low	Low	Low	Low	**Low**
Daley	2018	**Moderate**	Low	**Moderate**	**Moderate**	Low	Low	**Moderate**
Hefni	1993	**Low/Moderate**	Low	Low	Low	Low	**Low/Moderate**	**Low**
Hizli	2012	Low	Low	Low	Low	Low	Low	**Low**
Karateke	2016	**Moderate**	Low	Low	Low	Low	Low	**Low**
Kavehmanesh	2004	**Low/Moderate**	Low	Low	Low	**Low/Moderate**	Low	**Low**
Malhotra	1989	**Moderate**	Low	Low	**Low/Moderate**	Low	Low	**Moderate**
Makvandi	2013	**Moderate**	Low	Low	Low	Low	Low	**Low**
Naderi	2004	**Moderate**	Low	Low	Low	Low	Low	**Low**
Ozturk	2011	**Moderate**	Low	Low	Low	**Low/Moderate**	Low	**Low**
Petherick	2014	**Moderate/Serious**	Low	**Moderate**	Low	Low	Low	**Moderate**
Rezk	2016	Low	Low	Low	Low	Low	Low	**Low**
Sakar	2016	Low	Low	Low	Low	Low	Low	**Low**
Sarafraz	2013	Low	Low	Low	Low	Low	Low	**Low**
Savitri	2018	Low	**Serious**	Low	Low	Low	Low	**Moderate**
Seckin	2014	Low	Low	Low	Low	Low	Low	**Low**
Shahgheibi	2005	**Low/Moderate**	Low	Low	Low	Low	Low	**Low**
Ziaee	2010	Low	Low	Low	Low	Low	Low	**Low**

Studies found to have a moderate or greater risk of bias in one or more domains are highlighted in bold

### Synthesis of results

**Fig. 2:**
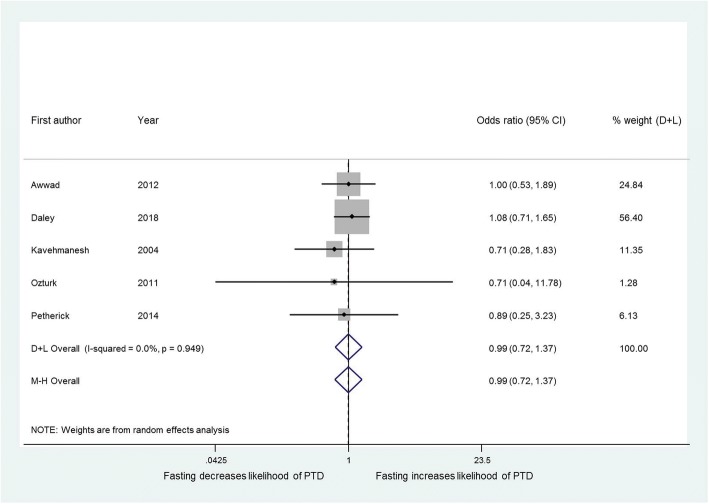
Effect of fasting on the likelihood of preterm delivery. Forest plot showing the effect of maternal fasting on preterm delivery

No studies presented data regarding perinatal mortality, and only two [10, 11] had data for SGA infants so meta-analysis was not performed. There was no significant effect of Ramadan fasting on the frequency of preterm delivery (OR 0.99, 95% CI 0.72 to 1.37) (Fig. 2); data were available on 5600 pregnancies from five studies [9, 10, 12, 13, 26] of which 1193 were exposed to Ramadan fasting. One study defined preterm delivery as < 38 weeks gestation so these data were not included [27]. Another study excluded preterm deliveries from the cohort [28].

**Fig. 3:**
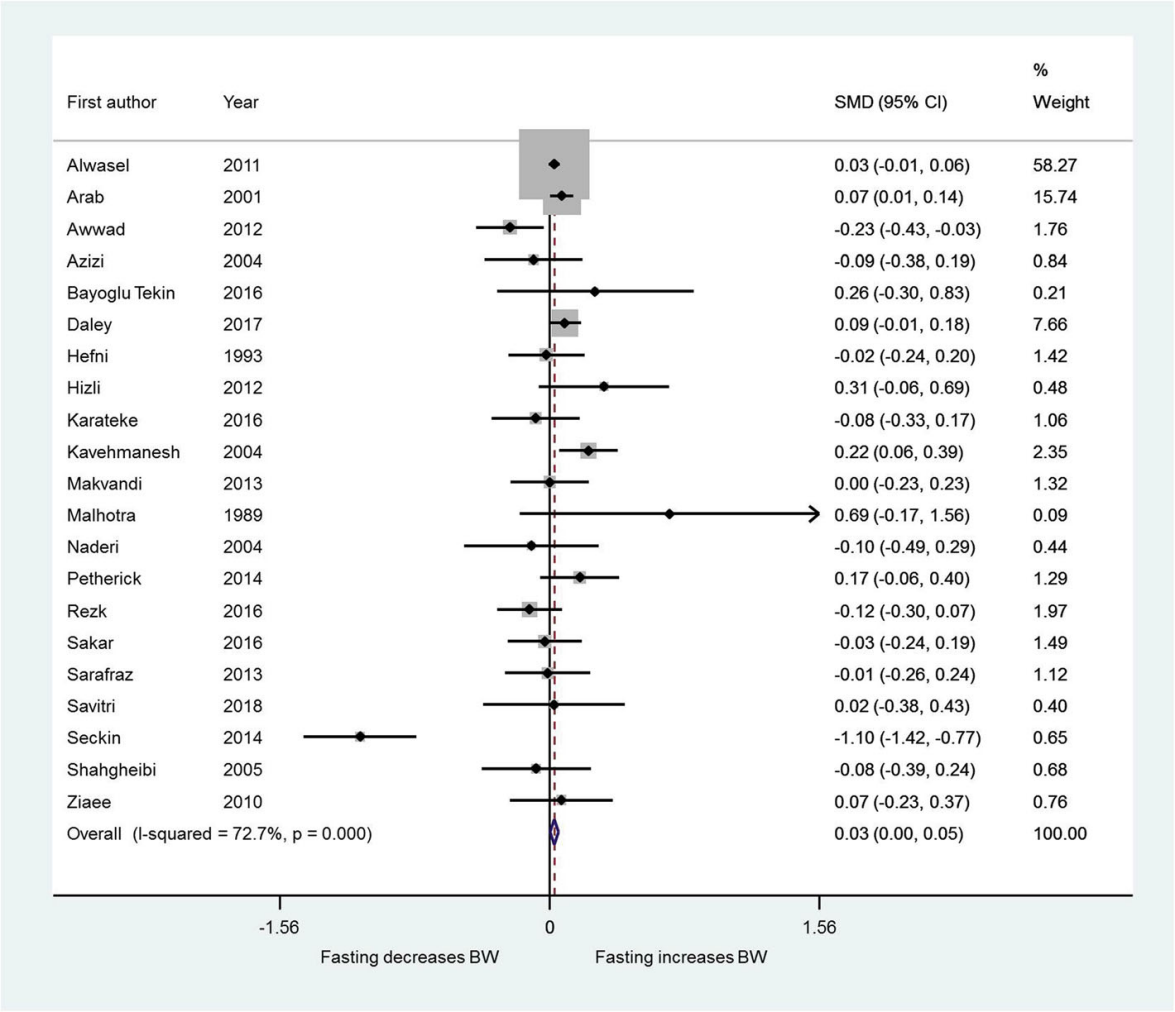
Effect of fasting on birth weight. Forest plot showing the effect of maternal fasting on birth weight as a continuous variable

All but one study [9] examined birth weight as a continuous variable; data were available on 31,441 pregnancies, of which 19,030 were exposed to fasting. There was no significant effect of maternal Ramadan fasting on birth weight (SMD 0.03, 95% CI 0.00 to 0.05) (Fig. 3). Three studies [29–31] presented mean results stratified by trimester in which fasting occurred, and an additional ten studies [11, 27, 32–39] were of third trimester exposure allowing a comparison to be performed; however no individual trimester showed a significant effect of fasting on birth weight and there was no difference between trimester groups (*p* = 0.99).

**Fig. 4:**
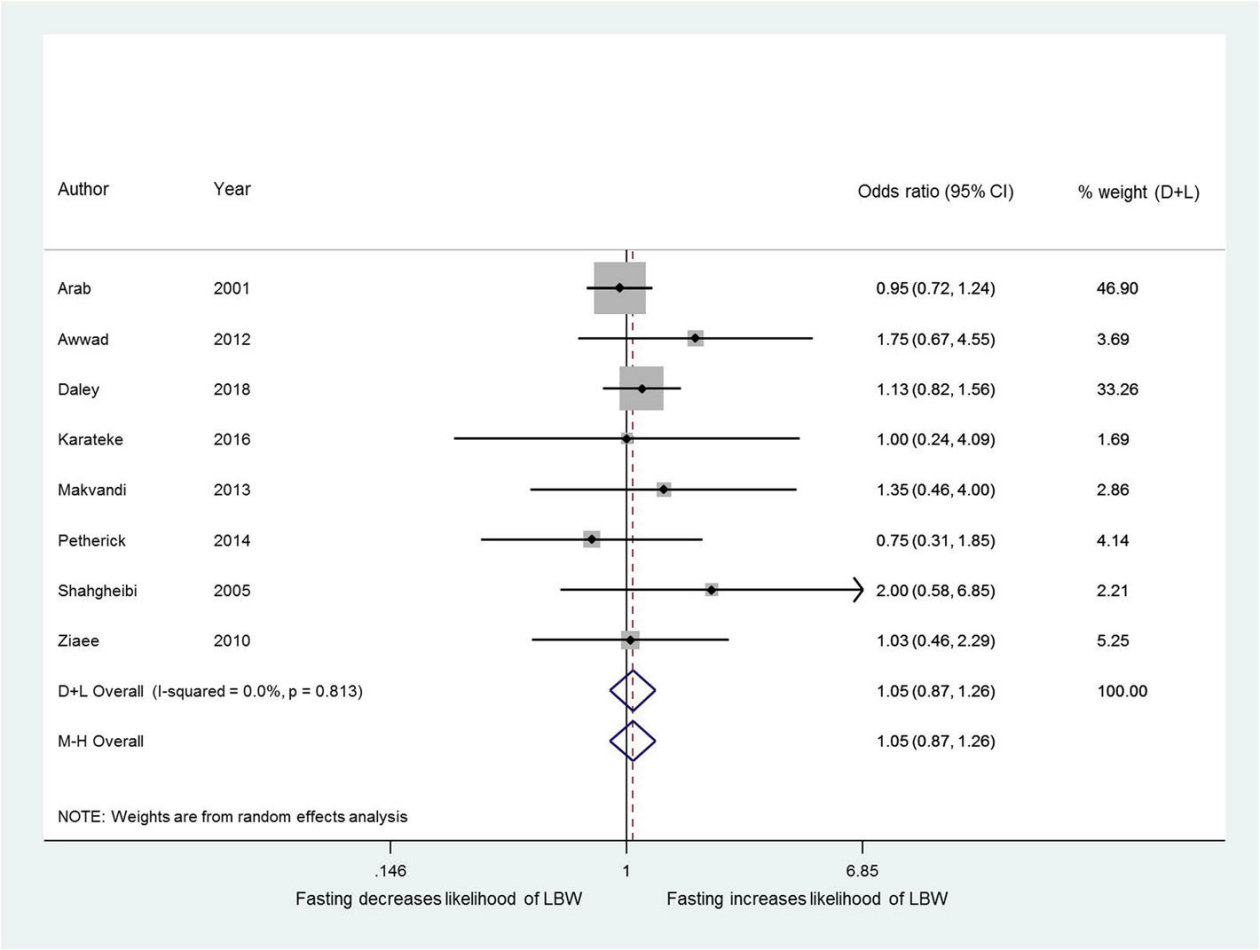
Effect of fasting on likelihood of low birth weight births. Forest plot showing the effect of maternal fasting on low birth weight (< 2500 g) births

Eight studies [10, 13, 26, 30, 31, 35, 39, 40] investigated the effects of maternal fasting on low birth weight (LBW); there were 11,080 births from these studies, of which 4344 were from mothers who fasted. Fasting did not significantly affect the proportion of LBW babies (OR 1.05, 95% CI 0.87 to 1.26) (Fig. 4). Three of these studies [30, 31, 40] stratified their data by trimester (*n* = 2411 first trimester fasting, *n* = 2571 second trimester, *n* = 2356 third trimester); there was no significant difference in the effect (*I*^*2*^ 0·0% *p* = 0.57).

**Fig. 5:**
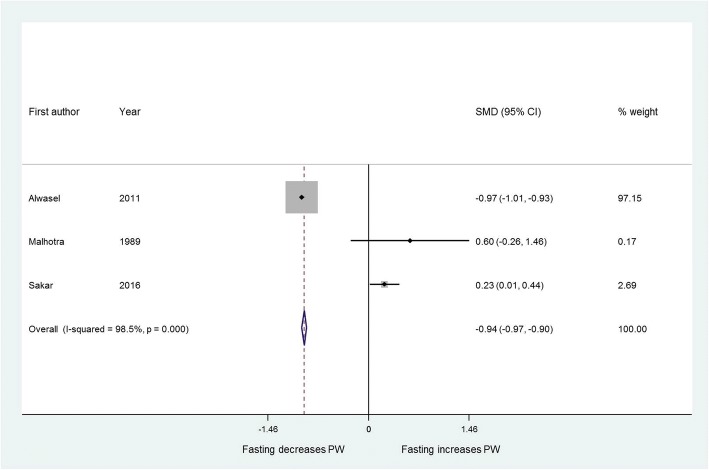
Effect of fasting on placental weight. Forest plot showing the effect of maternal fasting on placental weight as a continuous variable

Three studies comprising 17,986 pregnancies measured placental weight as an outcome [11, 29, 37]. Placental weight was significantly lower in fasting mothers (SMD -0.94, 95% CI -0.97 to − 0.90) (Fig. 5).

Two authors were contacted for information. One responded [33], providing clarification on study outcomes. No information was provided regarding discrepancies between numbers in tables and text in another paper [40]; data from the text were used as these were consistent with data reported in the abstract.

### Risk of bias of included studies

**Fig. 6:**
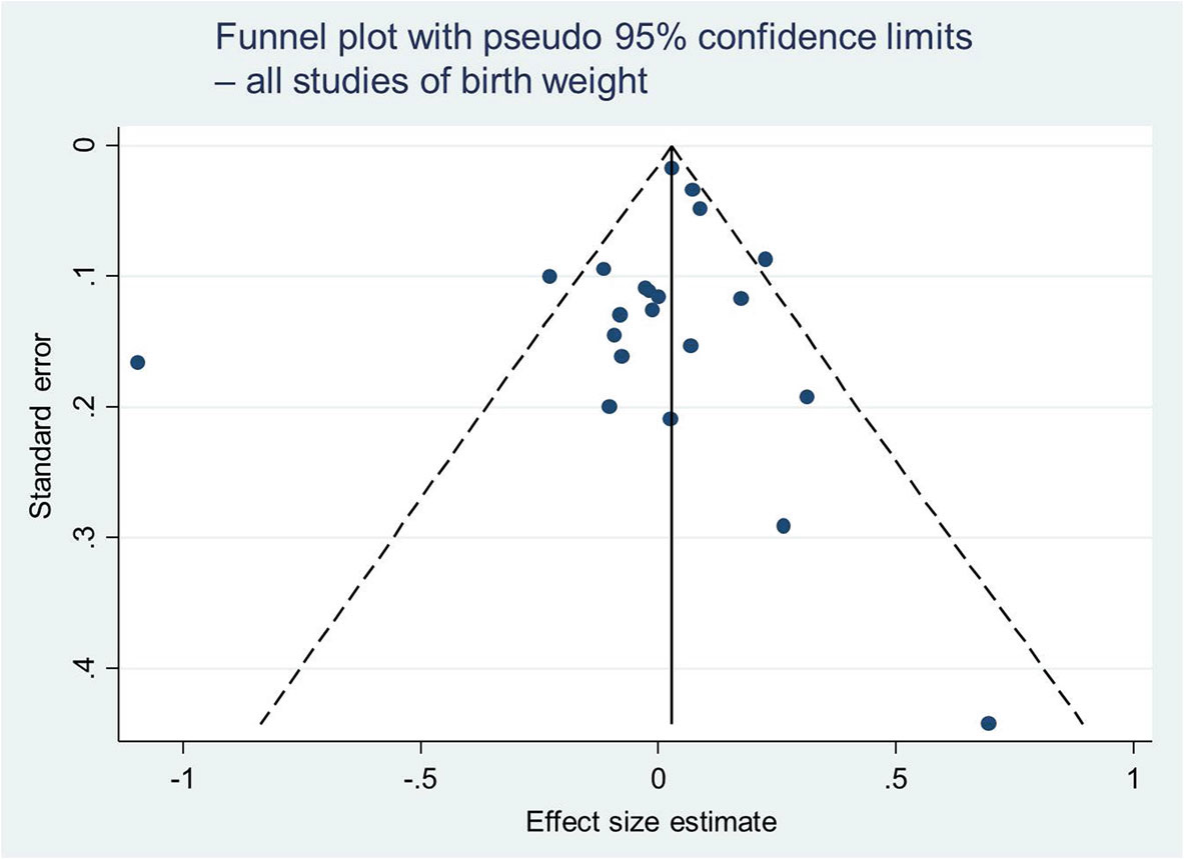
Funnel plot with 95% confidence limits. Funnel plot for all studies of birth weight

Egger’s test gave a value of *p* = 0.082 indicating that there was no significant influence of small study effects on our results (Fig. 6). No studies were assessed as having a high risk of bias so the analyses presented include all results. However, of the 31,441 pregnancies where birth weight was measured as an outcome, 17,626 were from one study [29]. A sensitivity analysis was performed to determine how much of an effect this study had on the overall result; without this study a SMD of 0.03 (95% CI -0.01 to 0.07) was obtained, still demonstrating no significant effect of fasting on birth weight.

## Discussion

Of our co-primary outcomes, only preterm birth had sufficient studies for meta-analysis which found no significant effect of Ramadan fasting. Data were available for some secondary outcomes: birth weight, placental weight and low birth weight; only placental weight was reduced by Ramadan fasting. However, this result was dominated by one study [29], which comprised 17,626 of 17,986 births for this outcome; one of the other two studies found a significant increase in placental weight [37]. There were insufficient data to perform meta-analysis for other outcomes, including: congenital abnormalities, gestational diabetes, hypertension, stillbirth and neonatal death. Stillbirth and neonatal death are arguably the most serious of these outcomes, yet due to the relatively small number of studies and their comparatively low incidences this study was underpowered to detect a difference. The paucity of data indicates a need for further large scale studies which report data on these rare but serious outcomes.

### Strengths and limitations

This study was strengthened in that it was carried out in accordance with a prospective protocol with pre-specified eligibility criteria and primary outcomes. This is the first meta-analysis to examine the effects of Ramadan fasting and provides a dataset that can be updated as the number of studies grows. It has also highlighted the current lack of data and identified research gaps to be addressed. This study was limited by the fact that due to resources only English language papers were included. Furthermore, other potential effects of Ramadan fasting may not have been included in our outcome measures. Other studies have shown effects of Ramadan fasting on fetal movement [41], maternal glucose levels [42] and various fetal growth indices [43]. Furthermore, our review did not examine whether fasting in the periconceptional period was associated with pregnancy outcome. As enduring effects of fasting and maternal undernutrition in this period have been shown in animal models [44, 45], this hypothesis merits further exploration.

The studies reviewed suggest that pregnant women who are well nourished may have nutritional reserves to support fetal adaptations during Ramadan fasting. However, longitudinal information on fetal growth was not available; the only feasible measure recorded was birth weight. Therefore, it remains to be established whether Ramadan fasting alters fetal growth patterns. Furthermore, there is little known about postnatal growth or growth and development in infancy and childhood.

Our literature search also identified papers that reported data on co-primary or secondary outcomes but presented the data in ways that could not be incorporated; Salleh [46] used linear regression to examine the effects of Ramadan fasting on birth weight and Boskabadi et al. [47] also looked at birth weight but presented data as medians and interquartile ranges; there was no control group in the study. One of our included studies [9] contained usable data for preterm delivery; however birth weight data were presented as medians and IQRs. Neither of these studies found differences between the control and fasting groups. Other studies were excluded because study groups were not sufficiently clear: Almond and Mazumder [48] acknowledged that not all women in their ‘fasting’ group fasted and that a large number of non-Muslims may also have been included. Cross et al. [49] defined maternal Muslim status (and assumed fasting status) based on the first three letters of maternal surnames.

Data showed significant heterogeneity for some outcomes. This variation may not relate to trimester of fasting, as when data were stratified by trimester there were no significant differences in the observed effect, although this may also represent a type 2 error as this meta-analysis may not have sufficient statistical power to detect such a difference. Only three studies presented usable data stratified by trimester of fasting for birth weight [29–31], of which one [31] found an association between trimester and mean neonatal weight. Alwasel et al. [29] showed significant associations in the second and third trimesters but not the first. Savitri et al. [28] performed regression analysis to investigate the effect of fasting trimester and found no significance, although they state that there was a trend towards lower birth weight with fasting, particularly in the second and third trimester. It may be that fasting later in pregnancy, when fetal growth is exponential, would be more likely to impact birth weight; further human studies are needed.

We were not able to investigate potential effects of Ramadan fasting length (in days) and duration (hours/day) due to limitations in available data. Duration of fasting was not documented by all studies and data were recorded in different ways; some studies stated the average number of fasting hours per day [9, 12, 32–34, 36] while others gave the upper [13] or lower [27] limits. In total, 16 included studies recorded the average number of fasting days (Table 1), but few papers stratified by number of days fasting so meta-regression could not be performed. However, only one paper that divided data by number of fasting days [40] found a significant difference in outcome: that birth weight following more than 20 days of fasting was significantly greater than that after fasting for 1–9 days. Makki [50] found no relationship between the number of fasting days and incidence of low birth weight. However, this paper could not be included in our analysis as there was no comparator group.

Another potential source of heterogeneity was geographical location of study. The majority of studies were from Asia and the Middle East (8 from Iran [12, 31, 32, 35, 39, 40, 51, 52], 6 from Turkey [9, 30, 33, 34, 37, 38], 2 from Egypt [27, 36], 1 from Indonesia [28], 1 from Saudi Arabia [29] and 1 from Lebanon [10]). Three included studies [11, 13, 26] were from the UK. Geographical location may alter the number of hours of fasting, and thus the physiological challenge on the developing fetus, as the timing of the daily fast is determined by sunrise and sunset.

Risk of bias is unlikely to account for the observed heterogeneity as overall risk of bias of the included studies was low, with only six studies [11, 13, 26, 28, 29, 40] judged to be at moderate risk of bias. The majority of bias was due to uncertainty of the trimester affected by fasting. Three studies were judged to be at serious risk of bias for individual domains: one paper was due to missing data [40], another for selection of participants [28], and the other due to classification of exposure [29]. Therefore, subgroup analysis for risk of bias was not conducted.

## Conclusions

This meta-analysis did not find any significant associations between Ramadan fasting and pregnancy outcome. Although studies were drawn from a large literature base, only a relatively small number met the inclusion criteria for analysis, limiting the breadth of robust conclusions. Until more definitive data are available, clinicians and other pregnancy healthcare providers cannot make firm recommendations that Ramadan fasting has no adverse consequences for mother or infant. Further observational studies of the effects of Ramadan fasting are required. Even if individual studies are not sufficiently large to determine differences in rare outcomes such as stillbirth or neonatal death, these should still be reported to facilitate subsequent meta-analysis. Additional studies are also needed to explore the origin of the considerable heterogeneity in observations; these should determine the effects of fasting in the periconceptional period, in different trimesters of pregnancy and whether geographical location, time of year and consequent duration of fasting alters the effect. Thus, well-designed studies investigating Ramadan fasting during pregnancy are needed to investigate the full impacts on maternal and fetal health, as well as to give potential fasting mothers an informed choice whilst addressing an issue that could have enduring public health consequences [53].

## Additional file

Additional file 1: EMBASE search strategy. (DOCX 13 kb)

## Abbreviations

CI: Confidence interval
CINAHL: Cumulative Index of Nursing and Allied Health Literature
EMBASE: Excerpta Medica database
EThOS: E-thesis online service
HMIC: Healthcare Management Information Consortium
LBW: Low birth weight
MEDLINE: National Library of Medicine journal citation database
OR: Odds ratio
PROSPERO: International prospective register of systematic reviews
ROBINS-I: Risk of bias in non-randomised studies - of interventions
SGA: Small for gestational age
SMD: Standardised mean difference
