# Supplementary material for: The effect of Ramadan fasting during pregnancy on perinatal outcomes: a systematic review and meta-analysis
Source: BMC Pregnancy Childbirth. 2018 Oct 25;18:421. doi: 10.1186/s12884-018-2048-y (PMC6202808; doi:10.1186/s12884-018-2048-y)
Supplement: Supplementary file 1 — EMBASE search strategy. (DOCX 13 kb) [file 12884_2018_2048_MOESM1_ESM.docx]

1. EMBASE; *ISLAM/; 12 results.

2. EMBASE; *MUSLIM/; 5 results.

3. EMBASE; ramadan.ti,ab; 972 results.

4. EMBASE; 1 OR 2 OR 3; 983 results.

5. EMBASE; exp PREGNANCY/; 562946 results.

6. EMBASE; exp PREGNANT WOMAN/ OR exp PREGNANT WOMEN/; 36159 results.

7. EMBASE; exp EXPECTANT MOTHER/; 210 results.

8. EMBASE; 5 OR 6 OR 7; 579151 results.

9. EMBASE; exp DIET RESTRICTION/; 91733 results.

10. EMBASE; fast*.ti,ab; 426367 results.

11. EMBASE; "intermittent fast*".ti,ab; 242 results.

12. EMBASE; 9 OR 10 OR 11; 469516 results.

13. EMBASE; 4 AND 8 AND 12; 64 results.

14. EMBASE; 13 [Limit to: Human and Publication Year 1975-2015]; 59 results.

15. EMBASE; 14 [Limit to: Human and English Language and Publication Year 1975-2015];
